# Subcellular localization of nucleolar protein 14 and its proliferative function mediated by miR-17-5p and E2F4 in pancreatic cancer

**DOI:** 10.18632/aging.204915

**Published:** 2023-07-27

**Authors:** Yong-Xing Du, Ying Zhou, Xiao-Hao Zheng, Yun-Jie Duan, Zong-Ting Gu, Ye-Feng Yin, Cheng-Feng Wang

**Affiliations:** 1 Department of Pancreatic and Gastric Surgery, National Cancer Center/Cancer Hospital, Chinese Academy of Medical Sciences and Peking Union Medical College, Beijing 100021, People’s Republic of China; 2 Department of Endocrinology, China-Japan Friendship Hospital, Beijing 100029, People’s Republic of China; 3 Department of Colorectal Surgery, National Cancer Center/Cancer Hospital, Chinese Academy of Medical Sciences and Peking Union Medical College, Beijing 100021, People’s Republic of China; 4 Shanxi Province Cancer Hospital/ Shanxi Hospital Affiliated to Cancer Hospital, Chinese Academy of Medical Sciences/Cancer Hospital Affiliated to Shanxi Medical University, Taiyuan 030013, Shanxi, People’s Republic of China

**Keywords:** pancreatic cancer, nucleolar protein 14, subcellular localization, miR17-5p, E2F4

## Abstract

Pancreatic cancer is one of the most lethal malignancies worldwide. Acquiring infinite proliferation ability is a key hallmark and basis of tumorigenesis. NOP14 is an identified ribosome biogenesis protein that plays potential roles in cell proliferation. However, the function and molecular mechanism of NOP14 remain ambiguous in most human cancers. In this study, we first investigated the subcellular localization and expression of NOP14 by multiple quantitative assays in pancreatic cancer. We confirmed that NOP14 was mainly localized in nucleolus in human pancreatic cancer cells. Then we studied the regulatory effects of this nucleolus protein on tumor cell proliferation *in vitro*. NOP14 was demonstrated to play a dominant pro-proliferation role in pancreatic cancer. Furthermore, we identified miR17-5p as a downstream target of NOP14. Transfection of miR17-5p mimics or inhibitors rescued the down- or upregulated effect of NOP14 on cell proliferation by regulating expression of P130. In addition, NOP14 induced expression of transcription factor E2F4 independent of miR17-5p/P130 signaling, which simultaneously activated a set of targeted genes, such as CCNE1, PIM1, AKT1 etc., to promote tumor proliferation. These findings might provide novel insights for better understanding the diverse function of NOP14 in human malignancies to develop new strategies for targeted therapy.

## INTRODUCTION

Pancreatic adenocarcinoma (PDAC) ranks the fourth leading cause of death in human malignancies [1]. Due to its insidious onset, rapid progression, and resistance to most current therapies, including radiotherapy, chemotherapy and targeted immunotherapy, the prognosis of patients with PDAC has not being improved significantly for decades. Moreover, PDAC is expected to become the second lethal cancer up to 2030 with annually increasing incidence and mortality [2, 3]. Therefore, it is urgent to develop new therapeutic targets or biomarkers to improve the current clinical dilemma.

In humans, the nuclear protein 14 (NOP14) gene is located on chromosome 4p16.317. Previous studies have shown that NOP14 participates in ribosome biosynthesis by affecting the processing and maturation of ribosomal RNA precursors [4]. To date, few studies have addressed the relationship between abnormal expression of NOP14 and tumor development. Zhou et al. first reported that NOP14 facilitates pancreatic cancer progression [5]. Our previous study also showed that NOP14 was significantly upregulated and associated with poorer prognosis in patients with pancreatic cancer [6]. However, Lei et al. showed that NOP14 inhibits the progression of breast cancer [7]. Similarly, studies by Li et al. also support NOP14’s inhibitory function in melanoma [8]. These studies indicate that NOP14 has both tumor-promoting and tumor-suppressive functions and plays diverse roles in different cancer cell background. The subcellular localization of a protein plays an important role in its biological function. However, the subcellular localization of NOP14 in cancer cells has rarely been reported. In this study, we first used immunofluorescence and nuclear-cytoplasmic separation assay to assess the localization and expression of NOP14 in pancreatic cancer cells. Furthermore, we studied the effect of NOP14 dysregulation on cancer cell proliferation and investigated its potential mechanism. These results could provide more insights into revealing the functional mechanism of NOP14 in pancreatic cancer, which in turn might facilitate the discovery of new therapeutic targets or biomarkers for improving the prognosis of patients.

## RESULTS

### Subcellular localization of NOP14 in pancreatic cancer cells

**Figure 1:**
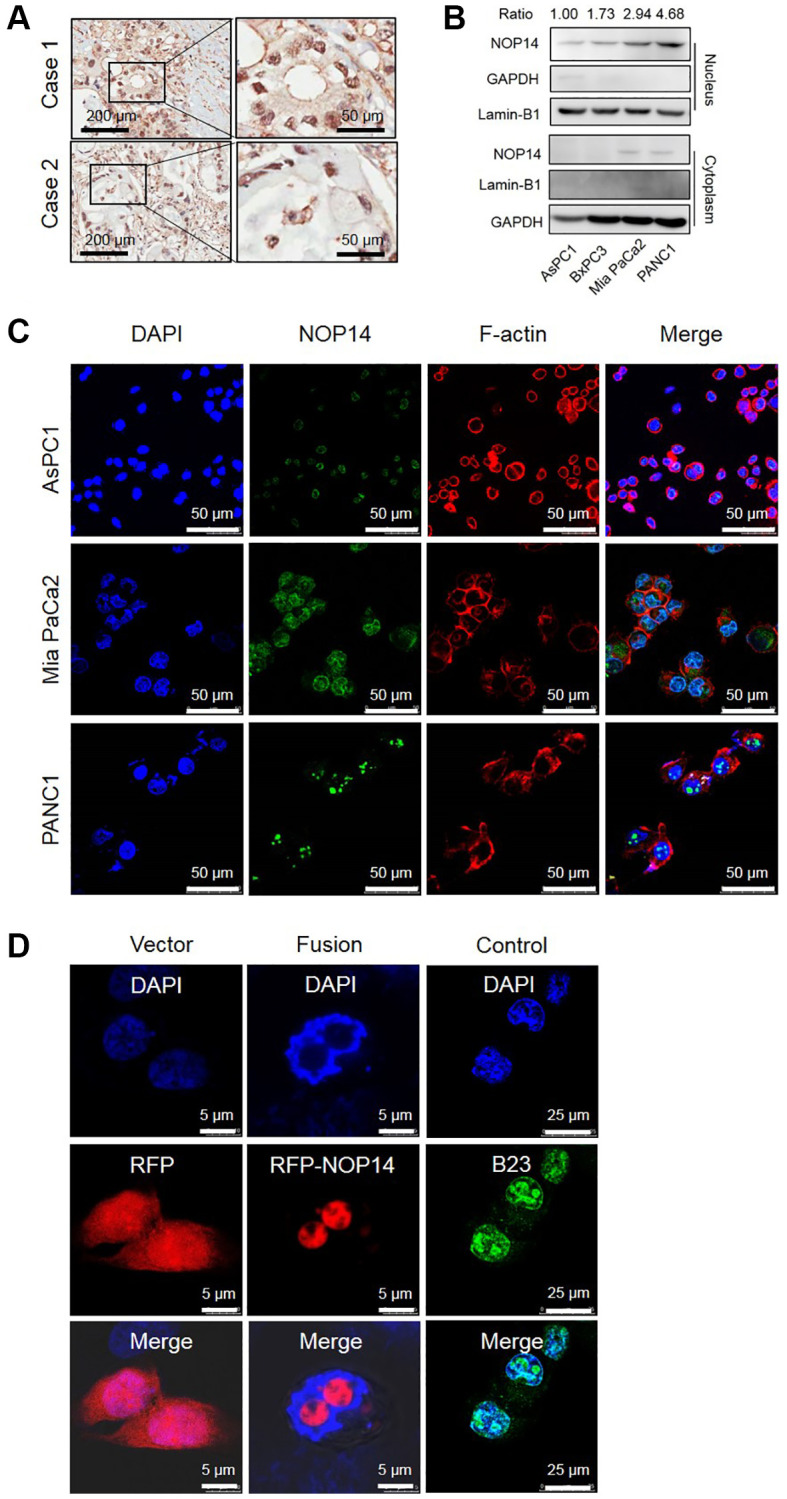
**Subcellular localization of NOP14 in pancreatic cancer cells.** (**A**) Immunohistochemical localization of NOP14 in pancreatic cancer tissues. (**B**) Western blot analysis of NOP14 in the nucleus and cytoplasm of different pancreatic cancer cell lines. Lamin B1 was used as the nuclear reference, and GAPDH was used as the cytoplasmic reference. Standardized grey ratio was shown on the top representing relative expression of NOP14 in nucleus. (**C**) Immunofluorescence analysis of NOP14 in pancreatic cancer cells under magnification 200×. Blue, DAPI-labeled nuclei; green, FITC-labeled anti-NOP14 antibody; red, phalloidin-labeled cytoskeletal F-actin; merge, superimposition of the above three images. (**D**) Immunofluorescence analysis of RFP-NOP14 expression in transfected PANC1 cancer cells. Blue, DAPI-labeled nuclei; red, RFP or RFP-NOP14; green, nucleolar protein B23; merge, superimposition of the above three images.

Previous studies showed that NOP14 was expressed in the nucleolus in yeast and was accordingly called nucleolar protein [9]. However, the functional localization of NOP14 in human malignant tumor cells has not been reported. In this study, we found that NOP14 is localized mainly in cell nuclei in pancreatic cancer tissues by immunohistochemical analysis (Figure 1A). Next, to validate this finding, we analyzed the expression of NOP14 in various pancreatic cancer cells in protein separation assay followed by Western blotting. Obviously, NOP14 was substantially detected in nucleus and its level was successively increased in AsPC1, BxPC3, Mia PaCa2 and PANC1 (Figure 1B), which was in lines with our previous report on total protein in relevant pancreatic cancer cell lines [10]. In contrast, NOP14 was rarely detected in the cytoplasm in any of those pancreatic cancer cell lines. Taken together, these results indicate that the NOP14 protein is localized mainly in the nucleus in pancreatic cancer cells.

To further study the nuclear localization of NOP14, we assessed its subcellular expressed location of NOP14 in pancreatic cancer cells by immunofluorescence analysis. As it was shown, NOP14 positive granules labeled by green fluorescence were visible with an obvious enrichment in specific areas of the nucleus (Figure 1C and Supplementary Figure 1). To confirm whether these specific areas were in the nucleolar region, we transfected exogenous RFP-NOP14 into PANC1 pancreatic cancer cells. The site of RFP-NOP14 expression was consistent with that of nucleolar protein B23, indicating that NOP14 was mainly expressed in the nucleolar with a little diffused in the nucleoplasm of pancreatic cancer cells (Figure 1D).

### Effects of NOP14 dysregulation on pancreatic cancer cell proliferation

To explore the effect of NOP14 dysregulation on cancer cell proliferation, we first analyzed the correlation between NOP14 level and cell proliferation in four commonly used pancreatic cancer cell lines. The expression of NOP14 increased successively in AsPC1, BxPC3, Mia PaCa2 and PANC1 (Supplementary Figure 2A), consistent with our previous findings. The proliferation assay showed a similar increase in proliferating ability in pancreatic cancer cell lines, indicating a positive correlation of proliferating ability with the expression of NOP14 (Supplementary Figure 2B).

**Figure 2:**
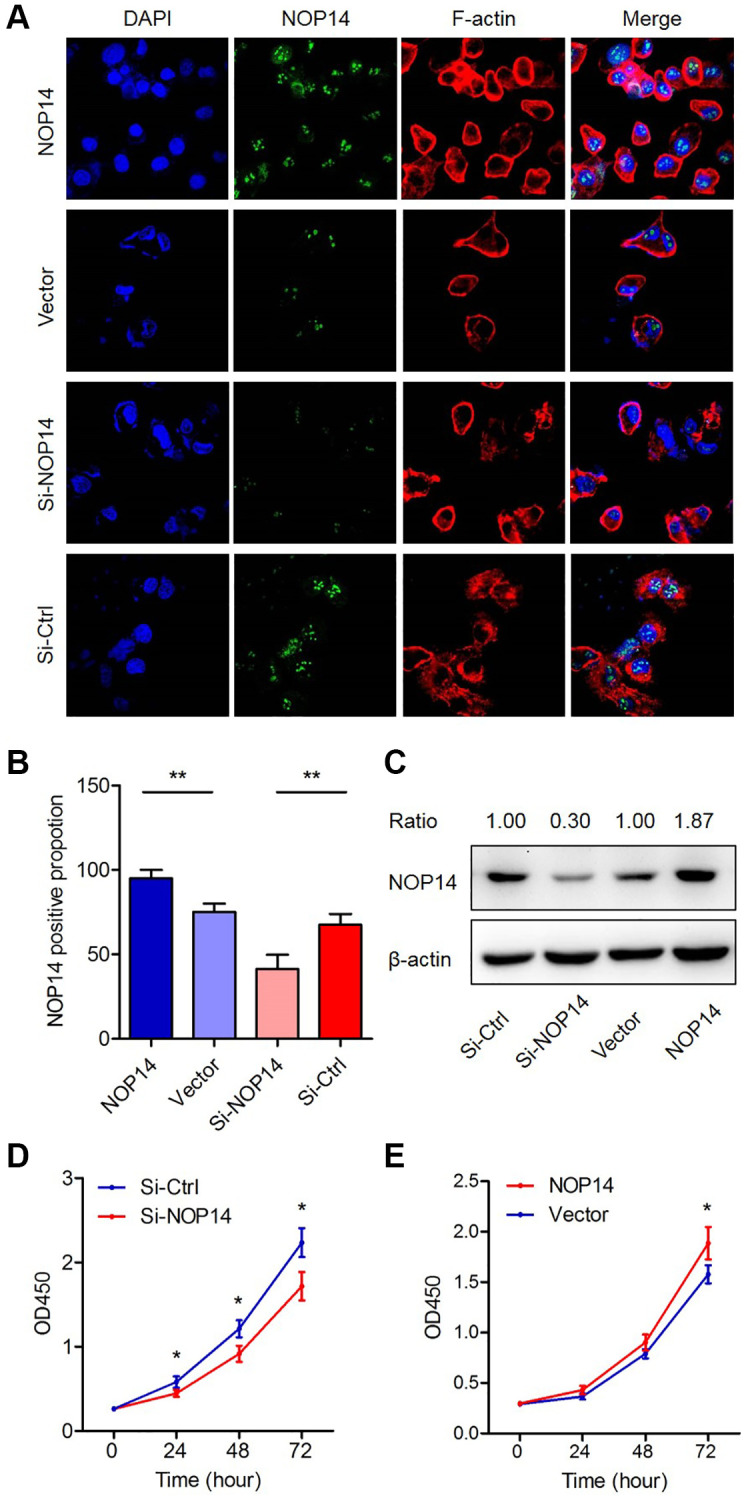
**Impacts of dysregulation of NOP14 on pancreatic cancer cell proliferation.** (**A**) Immunofluorescence analysis of NOP14 up- or downregulation in PANC1 pancreatic cancer cells. Magnification 100×. Blue, DAPI-labeled nuclei; green, FITC-conjugated anti-NOP14 antibody; red, phalloidin-labeled cytoskeletal F-actin; merge, superimposition of the above three images. (**B**) Proportion of NOP14-positive cells after up- or downregulation of NOP14, as assessed by fluorescence microscopy. (**C**) NOP14 expression level after up- or downregulation in PANC1 cancer cells, as determined by Western blot analysis with standardized relative gray ratio. (**D**) Effect of NOP14 inhibition on cell proliferation ability. (**E**) Effect of NOP14 overexpression on cell proliferation ability. ^*^P < 0.05, ^**^P < 0.01.

Next, we transfected pancreatic cancer cell with overexpression plasmid or interference siRNA to cause dysregulation of NOP14 and determined their effects on proliferation. Cell immunofluorescence experiments showed that NOP14 expression was significantly elevated or downregulated accordingly in the nucleus compared with that in the corresponding control group (Figure 2A and 2B). These results were also confirmed by Western blotting analysis (Figure 2C). Subsequent cell proliferation assays showed that the proliferation was enhanced or inhibited significantly after upregulation or downregulation of NOP14, respectively, in PANC1, indicating that NOP14 played a dominant role in promoting cancer cell proliferation (Figure 2D and 2E). We repeated these experiments in another pancreatic cancer cell line MiaPaCa2 and got similar results (Supplementary Figure 2C–2E). The above findings indicate that NOP14 promotes cell proliferation as an oncogene in pancreatic cancer.

### Identification of miR17-5p, a downstream target of NOP14

**Figure 3:**
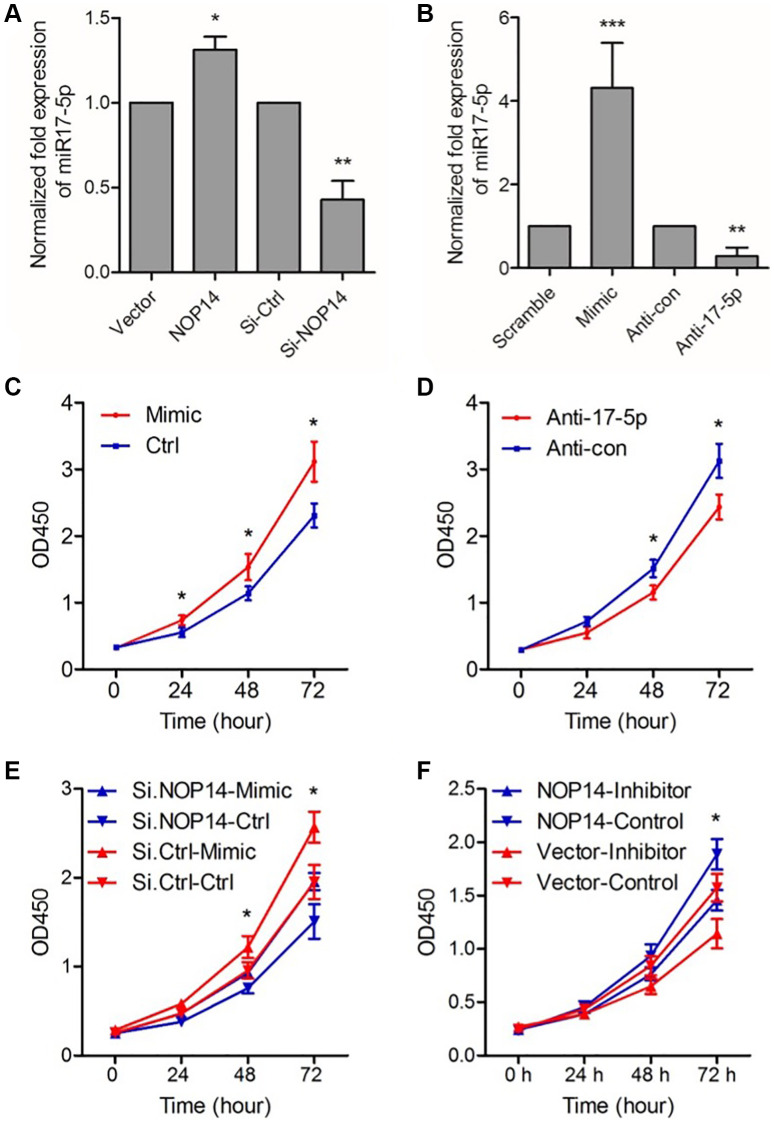
**miR17-5p mediates the promotive effect of NOP14 on the proliferation of PANC1 pancreatic cancer cell.** (**A**) Expression level of miR17-5p after up- or downregulation of NOP14, as determined by qRT–PCR. (**B**) Expression level of miR17-5p after transfection with the miR17-5p analog and control (scramble), inhibitor (anti-17-5p) and control (anti-con), as determined by qRT–PCR. (**C**) Cell proliferation ability after upregulation of miR17-5p expression by the miR17-5p analog. (**D**) Cell proliferation ability after downregulation of miR17-5p expression by anti-miR17-5p. (**E**) Cell proliferation ability after downregulation of NOP14 and upregulation of miR17-5p. (**F**) Cell proliferation ability after upregulation of NOP14 and downregulation of miR17-5p. ^*^P < 0.05, ^**^P < 0.01, ^***^P < 0.001.

To study the molecular mechanism of NOP14 in pancreatic cancer, we previously overexpressed and silenced NOP14 in PANC-1 cells and performed transcriptome sequencing to construct a map of NOP14 downstream target genes [6]. As a member of miR17~92 cluster, miR-17-5p was validated positively associated with NOP14 level in two pancreatic cancer cell lines with a noticeable significance. In this study, we confirmed that up- or downregulation of NOP14 induced a significant increase or decrease in miR17-5p expression (Figure 3A). To explore the regulation of cell proliferation by miR17-5p, we first overexpressed or silenced miR17-5p by transfecting a miR17-5p analog (MIC) or inhibitor (anti-17-5p), respectively, into PANC1 cells. It was shown that miR17-5p expression was significantly up- or downregulated accordingly (Figure 3B). Furthermore, the cell proliferation assay showed that upregulation of miR17-5p resulted in significantly enhanced cell proliferating ability (Figure 3C), whereas inhibition of miR17-5p led to remarkably decreased proliferation (Figure 3D). This pattern indicated that miR17-5p can significantly enhance the proliferation of pancreatic cancer cells.

Based on these results, we speculated that NOP14 might regulate the proliferation of pancreatic cancer cells by inducing miR17-5p expression. We designed a rescue experiment to verify this hypothesis. Firstly, the expression of NOP14 in PANC1 cells was downregulated by transfection of siRNA. Then, the expression of miR17-5p was upregulated by transfection of the miR17-5p analog (mimic). Cell proliferation assays showed reduced proliferation caused by inhibition of NOP14 could be rescued by upregulation of miR17-5p expression (Figure 3E). Similarly, we upregulated NOP14 and then downregulated miR17-5p in PANC1 cells. It was found that inhibition of miR17-5p expression significantly reversed the promotive effect of NOP14 upregulation on cell proliferation (Figure 3F). Taken together, these results suggest that miR17-5p mediates the regulatory effect of NOP14 on pancreatic cancer cell proliferation.

### Screening and verification of downstream target genes of the NOP14/miR17-5p pathway

**Figure 4:**
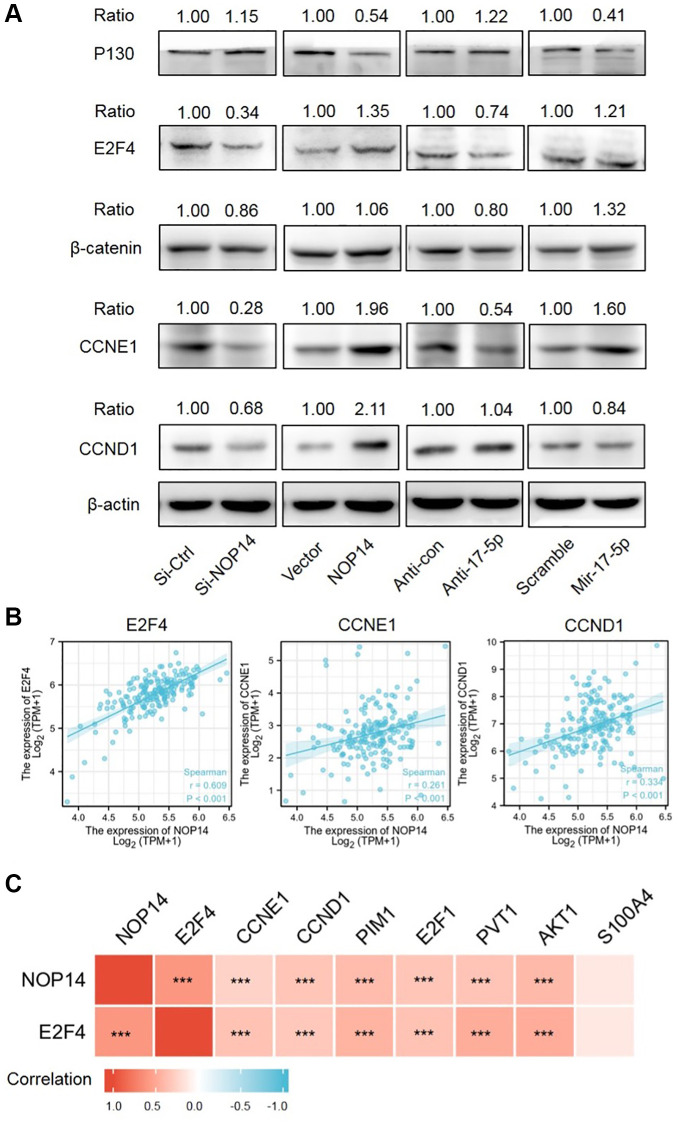
**Screening and verification of downstream targeted genes of the NOP14-miR17-5p pathway.** (**A**) Expression level of downstream targeted genes after up- or downregulation of NOP14 or miR17-5p, as determined by Western blot with standardized relative gray ratio. (**B**) Scatter plots showing significant positive correlation of E2F4, CCNE1, CCND1 with NOP14 in pancreatic cancer tissue from TCGA RNA seq data. (**C**) Heatmap diagram showing correlation of above targeted genes with NOP14 in pancreatic cancer tissue from TCGA RNA seq data. ^***^P < 0.001.

Previously, it was reported that miR17-5p could repress RBL2, also named P130, to release activating transcription factor E2F4 or CTNNB1, which promoted tumor progression by inducing activation of multiple targeted genes like CCND1 and CCNE1 etc., [10, 11]. Firstly, we studied the impacts of NOP14 or miR17-5p dysregulation on expression of these targeted genes. Western blotting assay showed overexpression of NOP14 or miR17-5p resulted in a marked reduction of P130 while inhibition of NOP14 or miR17-5p partly facilitated elevation of P130, indicating P130 was a downstream target of NOP14/miR17-5p signaling (Figure 4A). Meanwhile, our previous mRNA seq had identified that E2F4 was significantly positively corelated with NOP14 expression [10]. To validate this, we further analyzed the changes of E2F4 expression. Notably, NOP14 silencing induced a more noticeable inhibition of E2F4 than inhibitor of miR17-5p, and NOP14 overexpression led to a greater upregulation of E2F4 than mimic of miR17-5p, which suggested E2F4 was another target gene of NOP14 independent of miR17-5p. However, dysregulation of NOP14 or miR17-5p had quite less effect on expression of CTNNB1 than E2F4. Moreover, as targeted genes of E2F4, both CCND1 and CCNE1 showed an obvious upregulation or downregulation upon overexpression or suppression of NOP14. Taken together, E2F4 was another dominant target of NOP14 beyond miR17-5p/P130 signaling.

**Figure 5:**
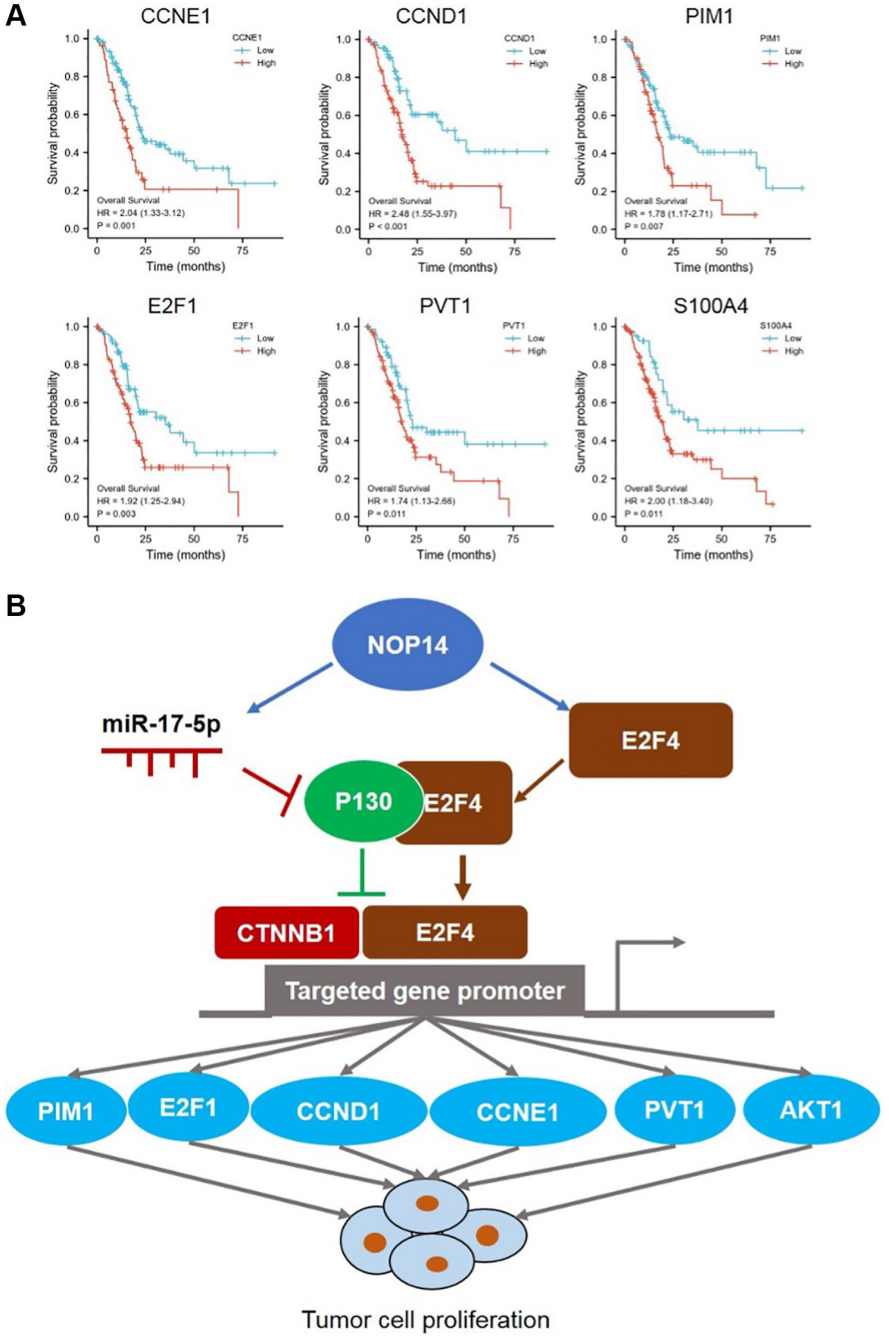
**Prognostic value of NOP14-miR 17-5p and -E2F4 pathway and their regulatory diagram.** (**A**) Kaplan–Meier survival analysis of NOP14 downstream targeted genes according to their expression in the TCGA pancreatic cancer dataset. The *P* value was obtained by Cox regression in R (version 3.6.3). (**B**) Schematic diagram showing the regulation of pancreatic cancer cell proliferation by the NOP14-miR 17-5p and -E2F4 pathway.

Next, we explored correlation between E2F4, CCNE1, CCND1 and NOP14 in pancreatic cancer tissue in TCGA database. It was found that NOP14 showed a stronger positive correlation with E2F4 (R = 0.609) than CCNE1 (R = 0.261) or CCND1 (R = 0.334) (Figure 4B). In addition, our previous transcriptome sequencing revealed a set of other potential targets of NOP14 except for E2F4, including PIM1, E2F1, AKT1, PVT1 and S100A4 etc., [6]. Interestingly, all these four genes are also the targeted genes of E2F4 according to ChIP-seq datasets from the ENCODE Transcription Factor Targets dataset. Similarly, correlation analysis further validated the positive regulatory relationship of PIM1 (R = 0.394), E2F1 (R = 0.333), AKT1 (R = 0.408) and PVT1 (R = 0.341) with NOP14, most of which was slightly weaker than their correlation with E2F4 (Figure 4C and Supplementary Figure 3A). To further validate the correlation of NOP14 with these target genes, we knocked down the expression of NOP14 in PANC1 and assessed impacts on their transcription. The result showed the expression of all those four genes were significantly inhibited after downregulation of NOP14 (Supplementary Figure 3C). This further suggested that NOP14 regulated these targeted genes by inducing the activation of E2F4. Furthermore, prognostic analysis showed higher expression of these proliferation activating genes except for AKT1 were significantly associated with poorer overall survival (Figure 5A and Supplementary Figure 3B).

In summary, our above findings suggested that NOP14 could induce activation of E2F4 by upregulating its expression or miR17-5p/P130 signaling to facilitate transcription of multiple targeted genes, including CCND1, CCNE1, PIM1, E2F1, AKT1 and PVT1 etc., which ultimately promoted tumor cell proliferation in pancreatic cancer (Figure 5B).

## DISCUSSION

The NOP14 gene is highly conserved in eukaryotes and encodes an 857-amino-acid protein [4]. The subcellular localization of proteins is extremely important for understanding the functions of proteins and designing drugs. In this study, the localization of NOP14 in pancreatic cancer cells was studied by immunofluorescence and protein separation quantitative analyses. We found that NOP14 was mainly localized in the nucleolus with only a small amount diffused in the nucleoplasm. It is well known that nucleolus plays an important role in ribosome formation and protein synthesis [12, 13]. As a nucleolar protein, NOP14 was reported to participate in processing and maturation of the ribosome [4, 14, 15]. To maintain a hyperproliferative phenotype, tumor cells require large numbers of ribosomes to synthesize proteins which is essential for various cellular physiological activities. Recent studies have shown that ribosomes are involved in DNA repair, cell cycle regulation and proliferation, and dysfunction of ribosomes can lead to tumor formation [16–18]. Many oncogenes and tumor suppressor genes, such as TP53, Rb, PTEN and c-myc, have been found to regulate the occurrence and development of tumors by disrupting ribosome biosynthesis [19–21]. In this sense, NOP14 might have an important role in tumor development and progression.

However, the function and molecular mechanism of NOP14 in most cancer remain unclear at present. Zhou et al. reported that inhibition of NOP14 reversed the malignant phenotype, indicating NOP14 functioned as an oncogene in pancreatic cancer [5]. In our previous studies [6, 22], NOP14 was found overexpressed in primary pancreatic cancer or metastases, which was significantly associated with poor prognosis. Moreover, we also revealed that NOP14 promoted the invasion and metastasis of pancreatic cancer *in vivo* and *in vitro* by increasing the stability of mutant p53. Inversely, studies in breast cancer and melanoma showed that NOP14 repressed tumor progression by regulating Wnt/β-Catenin signaling [7, 8]. Therefore, NOP14 has a dual function of promoting or inhibiting tumors and plays disparate roles in various cancers by regulating diverse downstream pathways.

In this study, we confirmed the impellent effect of NOP14 on cell proliferation in pancreatic cancer. Meanwhile, we identified miR17-5p as a downstream target to mediate pro-proliferative effect of NOP14 by inhibiting P130. miR17-5p is one of the mature microRNA clusters miR17~92, which are derived of MIR17 host gene and reported to function as oncogenic miRNA in most human malignancies [23, 24]. Our finding also supported the proliferative role of miR17-5p in pancreatic cancer, which was consistent with previous studies [25]. In addition, we found that dysregulation of NOP14 had little promotive effect on CTNNB1, also named β-Catenin, but induced a dominant abnormal expression of transcription factor E2F4 independent of miR17-5p/P130 signaling, which activated a set of targeted genes including CCND1, CCNE1, AKT1 etc. E2F4 is known to be a critical member of the transcription factors E2F family, which physically interact with the RB family proteins (RB, p130, and p107) at the transactivation domain to prevent the recruitment of transcriptional machinery [26]. These tightly regulated interactions ensure that cell cycle genes are expressed at the appropriate cell cycle stages [27]. Our data indicated NOP14 could not only activate E2F4 by inducing miR17-5p upregulation to inhibit its repressor P130 but also facilitating its own transcription. Previously we demonstrated NOP14 induced accumulation of mutant p53 protein by stabilizing its mRNA in pancreatic cancer [10], it is interesting to speculate that NOP14 might regulate E2F4 expression by directly binding and stabilizing its mRNA similar to mutant p53, which deserves further more investigations in the future. We further validated correlations between NOP14 and those downstream targets in pancreatic cancer tissues and verified their good prognostic value. This might explain the oncogenic roles of NOP14 in pancreatic cancer. On the other hand, there were also some discordances in this study. For example, we found that CCND1 showed inconsistent changes under overexpression of NOP14 and miR17-5p mimic, or transfection of NOP14 siRNA and miR17-5p inhibitor. This might be attributed to the direct silencing function of miR17-5p on CCND1, as CCND1 was demonstrated as one target of miR17-5p.

In conclusion, we reported the nucleolus localization and pro-proliferative effect of NOP14 by simultaneously activating downstream miR17-5p and E2F4 signaling in human pancreatic cancer. These findings might provide novel insights for better understanding the dual function of NOP14 in human malignancies to develop new strategies for targeted therapy.

## MATERIALS AND METHODS

### Clinical specimens and cell lines

Resected PDAC tissues from three patients were collected as described previously [9]. Human pancreatic cell lines were purchased from ATCC. The PANC-1 and MIA PaCa-2 human pancreatic cancer cell lines were maintained in Dulbecco’s modified Eagle’s medium, while ASPC-1 and BXPC-3 cells were maintained in RPMI 1640 medium (HyClone, USA). Both media were supplemented with 10% fetal bovine serum (FBS, HyClone, USA), 100 U/mL penicillin, and 100 mg/mL streptomycin at 37°C in a humidified atmosphere of 5% CO_2_.

### Immunohistochemistry (IHC)

IHC was conducted to measure NOP14 expression using an anti-NOP14 (Sigma–Aldrich, USA) antibody as described previously [9].

### Protein extraction

The process for protein extraction was as follows (OriGene, China): Pancreatic cancer cells were washed with cold phosphate-buffered saline (PBS) and dried. Then, 300 μL of cold extraction solution A was added to 30 μL of the precipitate, mixed by vortexing, and incubated on ice for 30 min with shaking. The samples were centrifuged at 1,200 × g for 5 min at 4°C. The supernatant comprised the cytoplasmic components, which were removed by pipetting and transferred into another precooled clean centrifuge tube and stored in a refrigerator. The precipitate was washed once with PBS and was then centrifuged at 2,000 × g for 5 min at 4°C. The supernatant was discarded. The resulting precipitate comprised the nuclear components and was resuspended in 200 μL of storage solution B and stored for later use.

### Cellular immunofluorescence analysis of NOP14

The process was as follows: (1) Cells were plated on specific aseptic round glass slides and cultured in a 12-well plate. When the cells were 40–50% confluent, the medium was removed, and the cells were washed with 200 μL of PBS. (2) A 200 μL volume of 4% paraformaldehyde was added to each well, incubated at room temperature (RT) for 15 min, and washed 3 times with 200 μL of PBS with gentle shaking. (3) To permeabilize the cell membrane, 0.5% Triton X-100 solution was added dropwise and incubated for 10 min, and the cells were then washed 3 times with 200 μL of PBS with gentle shaking. (4) A 200 μL volume of blocking solution (goat serum) was added and incubated at RT for 30–60 min. (5) The blocking solution was removed by aspiration, and 100 μL of the primary antibody was added and incubated in a wet box at 4°C overnight. (6) The cells were washed 3 times with 200 μL of PBS with gentle shaking, and the secondary antibody (FITC-conjugated, OriGene, China) was added and incubated at RT for 2 h. (7) The cells were washed 3–5 times with 200 μL of PBS with gentle shaking, stained with 4′,6-diamidino-2-phenylindole (DAPI) for 2 min, and washed 3 times with 200 μL of PBS with gentle shaking. (8) Anti-fade agent was added, and the slides were sealed, observed under a fluorescence microscope using a 10× eyepiece and 4×, 10×, and 20× objectives, and photographed. The rate of positive NOP14 staining was calculated as the percentage of positive cells in each field of view; at least three fields of view were analyzed per group of cells.

### Cell transfection experiments

For NOP14 overexpression, the full-length human NOP14 cDNA was amplified by polymerase chain reaction (PCR) and inserted into the pcDNA3.1 vector. NOP14 was downregulated by transfection of a small interfering RNA (siRNA). miR17-5p was upregulated by transfection of its analog (mimic) and downregulated by transfection of its inhibitor. Cells transfected with empty vector were used as controls. For the cell transfection experimental protocol, please refer to the instructions of Invitrogen Lipofectamine 2000/RNAiMAX. Cells were cultured at 37°C in a 5% CO_2_ incubator. After 48 h of transfection, cells were harvested for quantitative reverse transcription–PCR (qRT–PCR) and Western blot analyses. In the rescue experiment, the NOP14 overexpression plasmid or siRNA was transfected first, and then the miR17-5p inhibitor or analog was transfected 24 h later.

### Cell proliferation assay

Cell proliferation assays were performed using a CCK-8 kit to determine effects on pancreatic cancer cell proliferation. First, cells were harvested by trypsin digestion, resuspended, and counted, and the cell density was adjusted. Then, cells were seeded at 1,000 cells/well in a 96-well plate and incubated at 37°C in 5% CO_2_. After 24, 48, 72, and 96 h, the culture medium was discarded, and CCK-8 solution (10 μL/well) was added. Cell proliferation was assessed based on the optical density at 450 nm (OD450) as measured in a microplate reader (Bio-Tek, USA) after 2.5–3 h of incubation. At least three independent experiments were performed.

### Western blot analysis

Cells were lysed using ice-cold mammalian radioimmunoprecipitation assay (RIPA) buffer (Applygen Technologies Inc., China) containing a protease inhibitor cocktail and phenylmethylsulfonyl fluoride (PMSF). Proteins were quantified by a bicinchoninic acid (BCA) protein assay (Thermo Fisher Scientific, USA). Equal amounts of protein samples were separated by sodium dodecyl sulfate–polyacrylamide gel electrophoresis (SDS–PAGE), transferred onto a polyvinylidene difluoride (PVDF) membrane (Thermo Fisher Scientific, USA), and incubated with 10% nonfat milk overnight at 4°C. After washing three times with PBS containing Tween 20 (PBST), the membrane was incubated with the following primary antibodies for 1 h at RT: anti-NOP14 (Sigma–Aldrich, USA), anti-P130 (Abcam, USA), anti-β-Catenin, and anti-Cyclin D/E (Cell Signaling Technology, USA). After washing three times with PBST, the membrane was incubated with horseradish peroxidase (HRP)-conjugated goat anti-rabbit IgG H & L secondary antibodies at a 1:10,000 dilution. The membrane was rinsed, and protein bands were visualized using an enhanced chemiluminescence detection kit (Millipore, USA).

### Quantitative PCR (qPCR)

Total RNA was isolated from cultured cells using TRIzol reagent (Invitrogen, USA) and quantitated using a NanoDrop ND-1000 spectrophotometer (PeqLab, Germany). The reaction system was established in accordance with the instructions of an Invitrogen M-MLV reverse transcription kit. cDNA was obtained by reverse transcription using a TaKaRa RNA PCR kit (AMV) Ver. 3.0. The expression levels of candidate genes and GAPDH were determined by qRT–PCR using SYBR Green PCR Master Mix (Invitrogen, USA) in an ABI 7900HT Real-Time PCR System (Applied Biosystems, USA). qRT–PCR analysis of miR17-5p was performed using U6 as the internal reference. The thermal cycling conditions for PCR were as follows: 95°C for 10 min, followed by 40 cycles at 95°C for 15 s and 60°C for 1 min. All reactions were analyzed in triplicate. GAPDH mRNA was used as the endogenous control to analyze differences in RNA and cDNA levels within samples. Differences in the mean threshold cycle (Ct) values between the candidate mRNAs and GAPDH mRNA, indicated by ΔCt, were calculated to correct for differences in the amounts of extracted mRNA and the efficiency of the reverse transcription. The relative amount of each target mRNA was calculated as ΔΔCt and expressed as the fold change compared with a negative control sample. The primers used for amplification were described in previous study [9].

### Statistical analysis

All statistical analyses were performed by using SPSS 16.0 software (SPSS Inc.,). The relative expression levels of the target genes were quantified and analyzed using GraphPad Prism 5.0 software (GraphPad Software Inc., USA). All experiments were performed in triplicate. Data were reported as the mean ± standard deviation (SD) values. Data were analyzed using Student’s *t* test (two-tailed) for two-group comparisons and one-way analysis of variance for multiple-group comparisons. For molecular correlation analysis, Spearman correlation index R were calculated in R (version 3.6.3) with visualization of scatter diagram by ggplot2 (version 3.3.3). For Kaplan–Meier survival analysis, the *P* value was obtained by Cox regression in R (version 3.6.3). *P* values < 0.05 were considered statistically significant.

### Data availability

The data supporting this study can be obtained from the public databases or are available on request from the corresponding author.

## Supplementary Materials

Supplementary Figures
