# Supplementary material for: Subcellular localization of nucleolar protein 14 and its proliferative function mediated by miR-17-5p and E2F4 in pancreatic cancer
Source: Aging (Albany NY). 2023 Jul 27;15(14):7308–23. doi: 10.18632/aging.204915 (PMC10415562; doi:10.18632/aging.204915)
Supplement: Supplementary Figures [file aging-15-204915-s001.pdf]

SUPPLEMENTARY FIGURES

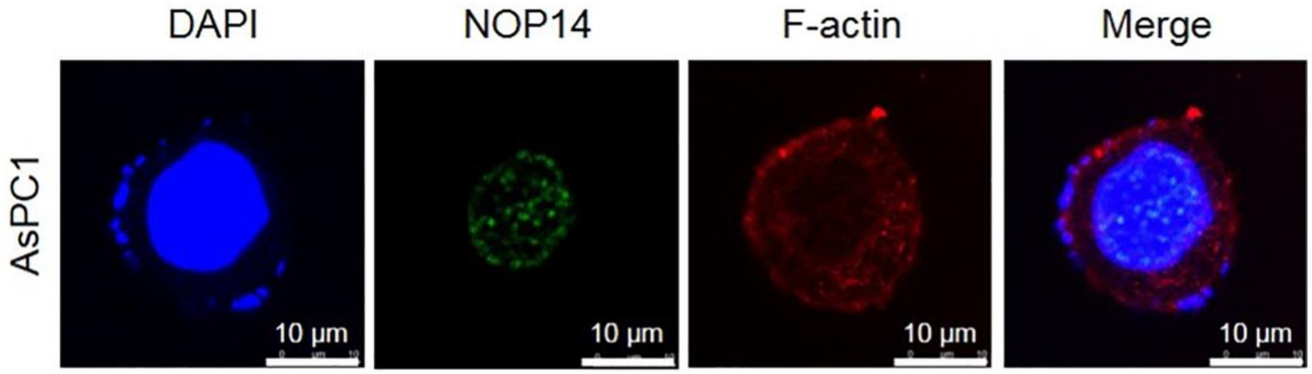

Supplementary Figure 1. Subcellular localization of NOP14 in AsPC1 pancreatic cancer cell. Immunofluorescence analysis of NOP14 in AsPC1 pancreatic cancer cell (under a 1,000× objective). Blue, DAPI-labeled nuclei; green, FITC-labeled anti-NOP14 antibody; red, phalloidin-labeled cytoskeletal F-actin; merge, superimposition of the above three images.

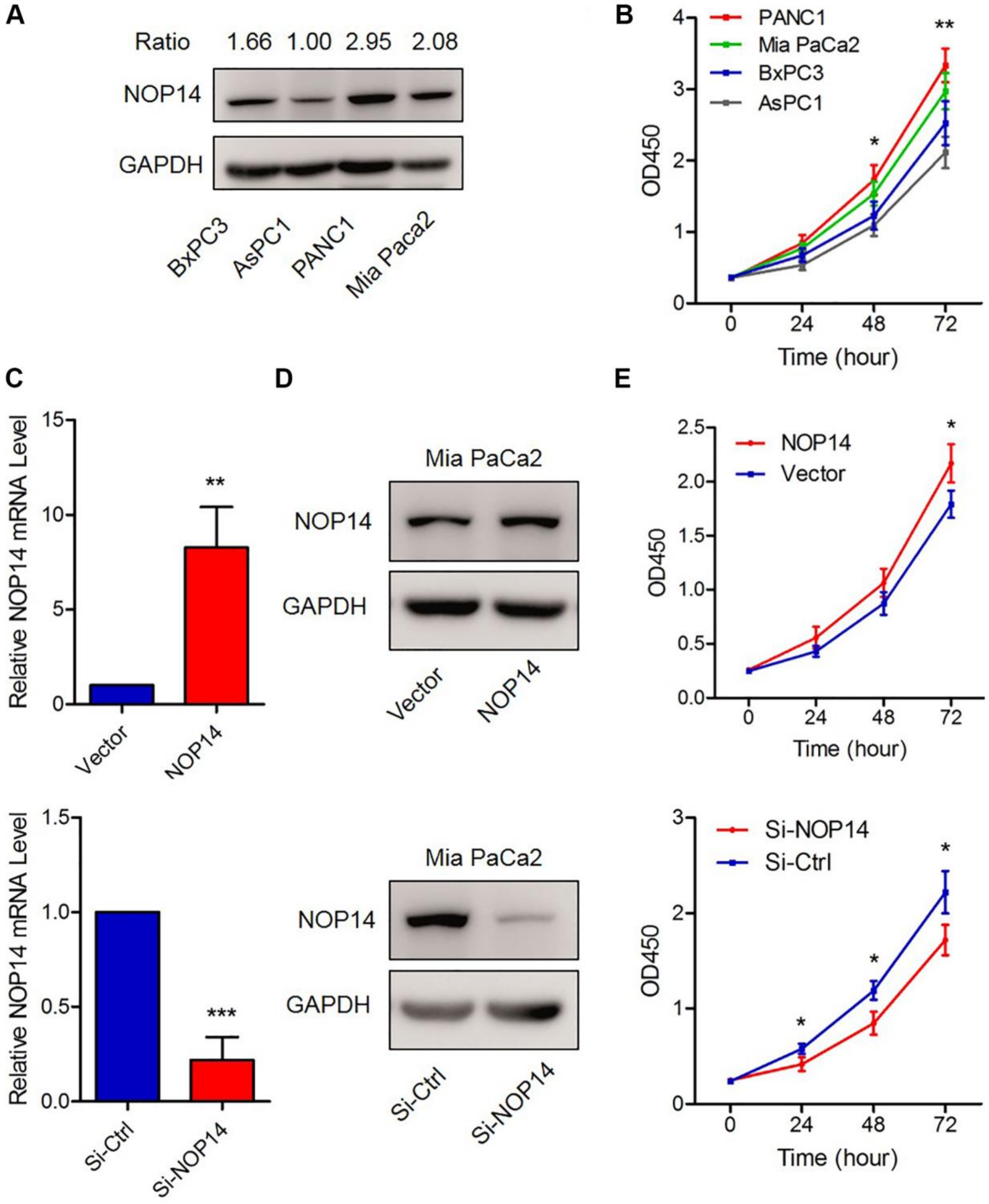

Supplementary Figure 2. Impacts of NOP14 expression on pancreatic cancer cell proliferation. (**A**) Expression level of NOP14 in four pancreatic cancer cell lines as evaluated by Western blot analysis with standardized relative gray ratio. (**B**) Proliferation ability comparison of four pancreatic cancer cell lines. (**C**) NOP14 expression level after up- or downregulation of NOP14 in Mia PaCa2 cells, as determined by qRT–PCR. (**D**) NOP14 expression level after up- or downregulation of NOP14 in Mia PaCa2 cells, as determined by Western blot analysis. (**E**) Proliferation ability of Mia PaCa2 cells after up- or downregulation of NOP14. $^{*}P < 0.05$, $^{**}P < 0.01$, $^{***}P < 0.001$.

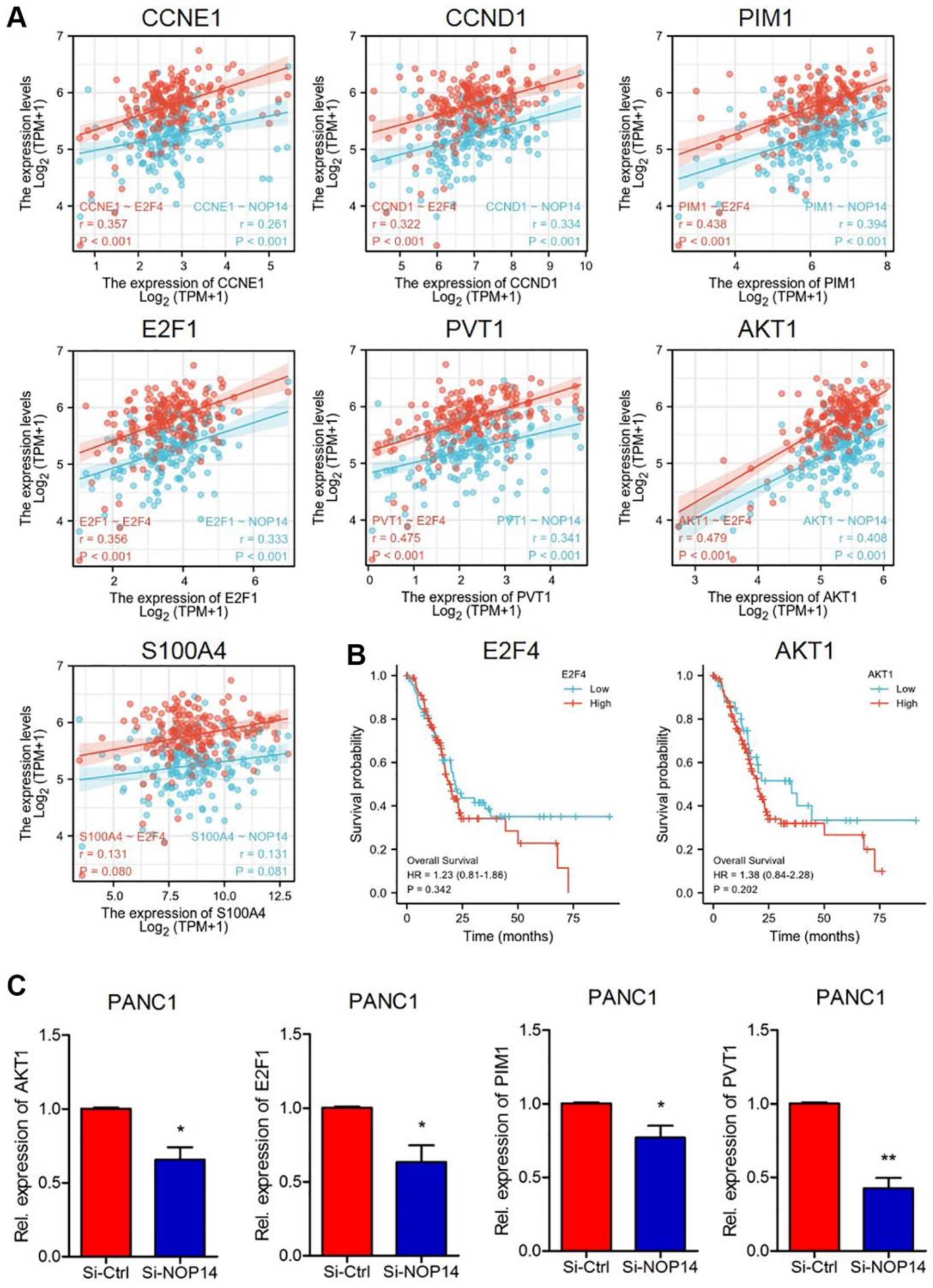

Supplementary Figure 3. Correlation and prognostic analysis of downstream targeted genes with NOP14 and E2F4. (**A**) Scatter plots showing correlation analysis of downstream targeted genes with NOP14 and E2F4 in pancreatic cancer tissue from TCGA RNA seq data. (**B**) Kaplan–Meier survival analysis of gene E2F4 and AKT1 according to their expression in the TCGA pancreatic cancer dataset. The *P* value was obtained by Cox regression in R (version 3.6.3). (**C**) Transcriptional Inhibition of target genes after downregulation of NOP14, as determined by qRT–PCR with Paired *t* test. $^{*}P < 0.05$, $^{**}P < 0.01$.
